# The effect of long-term amiodarone administration on myocardial fibrosis and evolution of left ventricular remodeling in a porcine model of ischemic cardiomyopathy

**DOI:** 10.1186/s40064-016-3249-3

**Published:** 2016-09-15

**Authors:** Anastasia Zagorianou, Meletios Marougkas, Stavros G. Drakos, Nikolaos Diakos, Panagiotis Konstantopoulos, Despina N. Perrea, Maria Anastasiou-Nana, Konstantinos Malliaras

**Affiliations:** 1 3rd Department of Cardiology, University of Athens School of Medicine, Laiko Hospital, 67 Mikras Asias Street, 11 527 Athens, Greece; 2 Laboratory for Experimental Surgery and Surgical Research “N.S. Christeas”, University of Athens School of Medicine, Athens, Greece

**Keywords:** Cardiac remodeling, Amiodarone, Myocardial infarction, Heart failure, Myocardial fibrosis

## Abstract

Amiodarone is effective in suppressing arrhythmias in heart failure patients. We investigated the effect of long-term amiodarone administration on myocardial fibrosis and left ventricular (LV) remodeling in a porcine model of ischemic cardiomyopathy. Eighteen infarcted farm pigs were randomized to receive long-term amiodarone administration for 3 months (n = 9) or conventional follow-up (n = 9). Evolution of LV remodeling over 3 months post-myocardial infarction was examined at tissue level (myocyte size, myocardial fibrosis and vascular density assessed by whole-field digital histopathology), organ level (LV structure and function assessed by echocardiography), and systemic level (BNP and MMP-9 levels). Long-term administration of the standard anti-arrhythmic doses of amiodarone was not associated with adverse effects on myocardial fibrosis and other features of adverse cardiac remodeling. This favorable safety profile suggests that long-term anti-arrhythmic therapy with amiodarone warrants further clinical investigation in the subpopulation of heart failure patients with significantly increased burden of arrhythmias.

## Background

Left ventricular (LV) remodeling is a complex pathophysiologic process characterized by changes at molecular, cellular and tissue level (Dixon and Spinale 2011). These changes result in cardiac dilatation and deterioration of LV function, and contribute to the onset and progression of heart failure (Pfeffer and Braunwald 1990). Amiodarone is an anti-arrhythmic drug that has proven to be effective in suppressing arrhythmias in heart failure patients, without increasing sudden cardiac death (Bardy et al. 2005). However, the impact of chronic amiodarone therapy on myocardial fibrosis and adverse LV remodeling remains unclear (Djandjighian et al. 2000; Tachikawa et al. 2005; Hu et al. 2004; Massie et al. 1996; Cleland et al. 1987; Hirasawa et al. 2009). We therefore sought to investigate the safety and efficacy of long-term amiodarone administration in a porcine model of ischemic cardiomyopathy. Given that amiodarone is known to promote interstitial pulmonary fibrosis (Jackevicius et al. 2011; Schwaiblmair et al. 2010; Papiris et al. 2010), the effect of chronic amiodarone administration on myocardial fibrosis was one of the main focuses of our investigation.

## Methods

### Surgical procedures and animal follow-up

A total of 18 farm pigs weighing ~30 kg were used in this experimental study. The experimental protocol was approved by the Veterinary Service of Athens and the ethical committee of the University of Athens, in accordance with national and EU legislation.

Animals were pre-anesthetized by intramuscular administration of ketamine (15–20 mg/kg), midazolam (0.5 mg/kg) and atropine (0.05 mg/kg). Auricular vein catheter placement was followed by induction of anesthesia with propofol (2 mg/kg). After induction of anesthesia, animals underwent transthoracic contrast echocardiography (Sonos 5500, Philips) and collection of blood samples. This was followed by endotracheal intubation and mechanical ventilation. All animals received mixtures of atmospheric air and oxygen (FiO_2_ = 30 %) with a tidal volume of 10–15 ml/kg of body weight and a respiratory frequency of 15/min. Throughout the experiment animals received intravenous infusion of propofol 10 mg/kg/h, fentanyl 20 g/kg and cisatracurium 0.2 mg/kg/20 min. The heart was accessed by lateral thoracotomy and myocardial biopsies were procured from the base of the LV. Afterwards, animals underwent induction of acute myocardial infarction (AMI) by permanent ligation of the left anterior descending coronary artery distal to the 1st diagonal branch, followed by closure of the chest. Animals were randomized into two groups: (a) animals in Group 1 (n = 9) received a loading dose of amiodarone (20 mg/kg IV during AMI) followed by daily p.o. administration of amiodarone (600 mg daily during the first 5 days post-MI, 300 mg daily during the following 3 months); (b) animals in Group 2 (control group, n = 9) underwent conventional follow-up. Animals were followed for 3 months. One month post-MI animals underwent transthoracic contrast echocardiography and collection of blood samples. Three months post-MI, animals underwent transthoracic contrast echocardiography, collection of blood samples and procurement of myocardial biopsies from the base of the LV and heart explantation. The explanted heart was sectioned into 1-cm-thick short-axis slices. Each slice was photographed with a digital camera, and infarct size was determined as the percentage of LV by manual tracing by a researcher blinded to treatment allocation.

### Histochemical stains and immunocytochemistry

Myocardial tissue immediately after its excision was fixed in 10 % buffered formalin and dehydrated in increasing concentrations of alcohol, then cleared through xylene and subsequently embedded in paraffin. The tissue sections were cut in 4-μm sections, collected and mounted on glass slides and prepared for various histochemical stains and immunohistochemistry.

#### *Masson’s trichrome*

Masson’s trichrome stain was used for collagen content evaluation. The stain was performed as previously described (Drakos et al. 2010).

#### *Periodic acid Schiff stain reaction (PAS)*

PAS stain reaction was used to evaluate cardiac myocyte size. The reaction is based on oxidation of certain tissue elements to aldehydes by periodic acid. The stain was performed as previously described (Drakos et al. 2010). PAS was selected for cardiomyocyte size evaluation because it offers sufficient cardiomyocyte basement membrane visualization.

#### *CD34 immunostaining*

Microvasculature evaluation was performed with immunostaining for endothelial cell protein CD34 using a mouse monoclonal anti CD34 antibody (Dako, Carpinteria, California). Immunohistochemistry experiments were performed using a peroxidase-conjugated streptavidin–biotin system and diaminobenzidine as a substrate. To achieve a high degree of reproducibility we avoided manual staining. Histochemical stains were performed using the automatic Artisan Special Stains System (Dako, Carpinteria, California) and the immunohistochemistry experiments were performed on the Autostainer (Dako, Carpinteria, California).

#### *Whole*-*field digital microscopy*

**Fig. 1:**
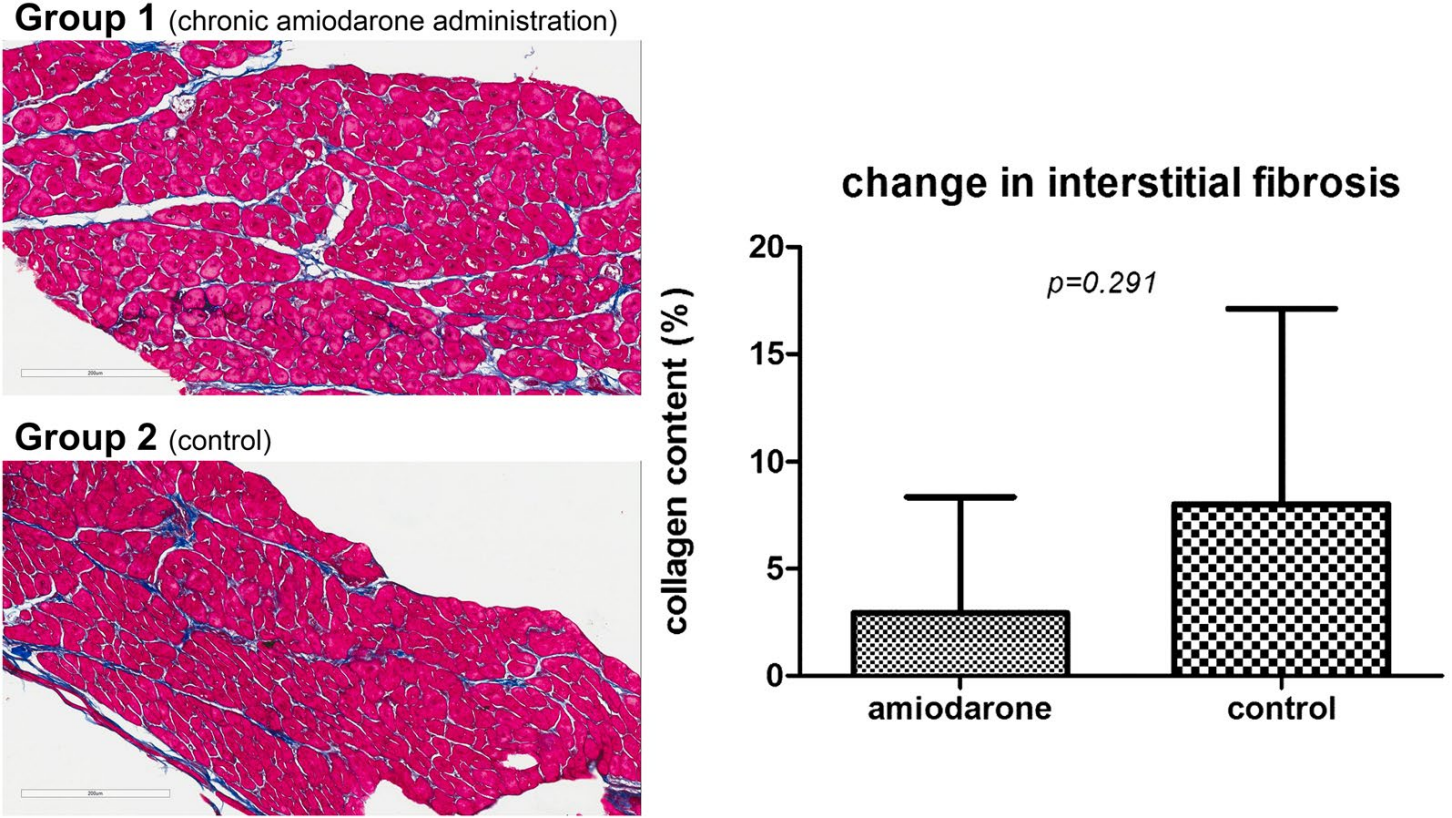
Chronic amiodarone administration post-myocardial infarction is not associated with increased myocardial interstitial fibrosis. Representative Masson’s trichrome-stained slides from an experimental animal randomized to receive chronic amiodarone administration (*top*) and a control animal (*bottom*). The absolute change in myocardial interstitial fibrosis (expressed as % of myocardium) between baseline (induction of myocardial infarction) and 3 months post-myocardial infarction is presented on the right. No increase in myocardial interstitial fibrosis was observed in animals receiving long-term amiodarone administration, compared to control animals

Advanced digital microscopy allowed examination of the entire heart tissue areas from the epicardium to the endocardium (Fig. 1), as described previously (Drakos et al. 2010). Whole-slide images were analyzed with the ScanScope XT system equipped with the ImageScope 10.0 image analysis algorithms (Aperio Technologies, Vista, California).

#### *Interstitial fibrosis evaluation*

We set the staining color threshold of the ImageScope 10.0 colocalization analysis algorithm to accurately identify collagen on the basis of its blue color as previously described (Drakos et al. 2010). Myocardial fibrosis was defined as the collagen content in the interstitial spaces and endomysial/perimysial spaces, including the collagen content around capillaries and vessels (expressed as % of total tissue analysis area).

#### *Microvasculature evaluation*

We used the ImageScope 10.0 microvessels analysis algorithm to distinguish endothelial cells from nonspecific staining of tissue by applying appropriate dark and light thresholds as previously described (Drakos et al. 2010). Only myocardial sections oriented in cross-section were analyzed. Microvascular density was defined as “microvessels/0.1 mm^2^”.

#### *Cardiomyocyte size evaluation*

Cardiomyocytes (40× magnification) were accepted for size measurement if they met the following criteria: (a) cellular cross-sections present (b) visible and round shaped nuclei located close to the cell center and (c) intact cellular basement membranes, as described previously (Drakos et al. 2010). The cross section area of 100 selected cardiomyocytes per sample was calculated by our digital histopathology system and then averaged.

### Assessment of the levels of circulating BNP and MMP9

The levels of circulating BNP and MMP9 were measured as biochemical markers of adverse remodeling (Fertin et al. 2012) at baseline and at 1 and 3 months post-MI. BNP and MMP9 concentration was measured in serum samples with the ELISA method, using commercially-available kits (USCN Life Science Inc).

### Statistical analysis

Continuous variables are presented as mean ± standard deviation. Normality of data was verified by the Kolmogorov–Smirnov test. Differences between the two groups were tested using independent samples *t* test. All tests were 2-sided. No multiplicity adjustment for multiple comparisons was performed. A *p* value of <0.05 was considered statistically significant.

## Results

### Adverse events and mortality

Nine animals were randomized to the amiodarone group and 9 animals to the control group. Six animals (four randomized to the control group and two to the amiodarone group) died during induction of AMI due to arrhythmia. One animal (randomized to the amiodarone group) died 1 week post-MI (due to surgical wound infection), and one (randomized to the amiodarone group) due to pulmonary edema. Five animals in each group completed the 3-month follow-up and were included in the analysis.

### Echocardiography

Absolute values of echocardiography-measured parameters are provided in Table 1. No differences were observed between groups at baseline and 1 month post-MI. Three months post-MI, animals randomized to the amiodarone group had significantly lower LV end-diastolic diameter compared to control animals (53.8 ± 4.0 mm vs. 60.0 ± 2.8; p = 0.029). No differences in LV end-systolic diameter and LV fractional shortening were observed at 3 months-post-MI between groups.

**Table 1:** Echocardiographic assessment of cardiac structure and function

Echocardiographic parameters	Group	Mean ± SD	p value
LVEDD (time 0)	Amiodarone	36.2 ± 1.6 mm	0.117
	Control	38.1 ± 1.8 mm	
LVESD (time 0)	Amiodarone	26.4 ± 3.2 mm	0.973
	Control	26.3 ± 2.0 mm	
LVFS (time 0)	Amiodarone	27 ± 8 %	0.363
	Control	31 ± 3 %	
LVEDD (1st month)	Amiodarone	47.7 ± 4.1 mm	0.591
	Control	46.3 ± 2.4 mm	
LVESD (1st month)	Amiodarone	36.3 ± 2.5 mm	0.163
	Control	31.4 ± 5.7 mm	
LVFS (1st month)	Amiodarone	23 ± 4 %	0.162
	Control	33 ± 11 %	
LVEDD (3rd month)	Amiodarone	53.8 ± 4.0 mm	*0.029*
	Control	60.0 ± 2.8 mm	
LVESD (3rd month)	Amiodarone	39.7 ± 4.1 mm	0.180
	Control	44.9 ± 5.7 mm	
LVFS (3rd month)	Amiodarone	26 ± 5 %	0.868
	Control	25 ± 10 %	

p value in italics indicates statistical significant differences between groups

*LVEDD* left ventricular end-diastolic diameter, *LVESD* left ventricular end-systolic diameter, *LVFS* left ventricular fractional shortening

### Histology

Macroscopic histological assessment of infarcted tissue did not reveal any morphometric differences between the two groups; infarct size (5.33 ± 0.57 % of LV in the amiodarone group vs. 5.33 ± 1.52 % of LV in the control group, p = 1.0) and infarct circumference (9.66 ± 4.5 % of LV circumference in the amiodarone group vs. 9.0 ± 1.7 % of LV in the control group, p = 0.823) were similar between groups. Microscopic histological assessments of cardiomyocyte size, vascular density and myocardial fibrosis are provided in Table 2. No differences in myocardial fibrosis, cardiomyocyte size, vascular density and myocardial fibrosis were observed between groups. Figure 1 shows representative Masson’s trichrome-stained slices from a treated and a control animal, and depicts the absolute change in myocardial fibrosis in the amiodarone (+3.2 ± 4.7 % of the LV) and the control group (+9.3 ± 10.7 % of the LV) between baseline (MI creation) and 3 months post-MI; no significant differences (p = 0.291) were observed between groups.

**Table 2:** Histological assessment of myocyte size, vessel density and myocardial fibrosis

Histological parameters	Group	Mean ± SD	p value
Myocyte size (baseline) (μm^2^)	Amiodarone	326.24 ± 58.30	0.197
	Control	264.60 ± 71.70	
Vascular density (baseline) (vessels/0.1 mm^2^)	Amiodarone	150 ± 100	0.073
	Control	340 ± 90	
Myocardial fibrosis (baseline) (%/area)	Amiodarone	8.44 ± 7.21	0.839
	Control	9.48 ± 5.57	
Myocyte size (3 months) (μm^2^)	Amiodarone	638.22 ± 259.70	0.874
	Control	607.31 ± 245.06	
Vascular density (3 months) (vessels/0.1 mm^2^)	Amiodarone	150 ± 100	0.150
	Control	310 ± 100	
Myocardial fibrosis (3 months) (%/area)	Amiodarone	11.60 ± 2.98	0.474
	Control	16.43 ± 14.12	

### Levels of circulating BNP and MMP9

The levels of circulating BNP and MMP9 were measured as biochemical markers of adverse cardiac remodeling (Fertin et al. 2012) and are provided in Table 3. No differences were observed between groups at any timepoint.

**Table 3:** Levels of circulating BNP and MMP9

Biochemical parameters	Group	Mean ± SD	p value
BNP (baseline)	Amiodarone	460.19 ± 489.93 pg/ml	0.345
	Control	206.89 ± 71.94 pg/ml	
MMP9 (baseline)	Amiodarone	3508.44 ± 964.32 ng/ml	0.387
	Control	4832.00 ± 3073.02 ng/ml	
BNP (1 month)	Amiodarone	242.59 ± 171.63 pg/ml	0.066
	Control	51.21 ± 26.65 pg/ml	
MMP9 (1 month)	Amiodarone	4363.77 ± 2404.90 ng/ml	0.406
	Control	6443.99 ± 4575.34 ng/ml	
BNP (3 months)	Amiodarone	341.90 ± 285.81 pg/ml	0.447
	Control	202.05 ± 216.96 pg/ml	
MMP9 (3 months)	Amiodarone	9181.24 ± 5077.68 ng/ml	0.714
	Control	7681.73 ± 6773.95 ng/ml	

## Discussion

Amiodarone is an anti-arrhythmic drug with proven efficacy in suppressing arrhythmias in heart failure patients, without increasing sudden cardiac death (Bardy et al. 2005). However, the impact of chronic amiodarone administration on the evolution of adverse LV remodeling requires further investigation, as experimental and clinical studies to date have yielded conflicting results (Djandjighian et al. 2000; Tachikawa et al. 2005; Hu et al. 2004; Massie et al. 1996; Cleland et al. 1987; Hirasawa et al. 2009). In addition, the effect of chronic amiodarone administration on myocardial fibrosis remains unclear; this issue merits further investigation, given that amiodarone is known to exert profibrotic effects in the lung parenchyma (Jackevicius et al. 2011; Schwaiblmair et al. 2010; Papiris et al. 2010). We therefore sought to investigate the effects of long-term amiodarone therapy on LV remodeling and myocardial fibrosis in a clinically-relevant model of ischemic cardiomyopathy.

We found that chronic amiodarone administration has no significant effect—either positive or negative—on the evolution of LV remodeling post-MI at the tissue (myocyte size, vascular density and interstitial fibrosis), organ (LV structure and function) and systemic level (levels of circulating BNP and MMP-9). While LV dilatation (as assessed by echocardiographic measurement of LVEDD) was attenuated in animals receiving amiodarone, this was not accompanied by concomitant beneficial changes in histological and biochemical indices of remodeling. In addition, since amiodarone is known to exert anti-beta adrenergic effects (Polster and Broekhuysen 1976; Nokin et al. 1983; Chatelain et al. 1995) (and given the fact that beta blockers were not administered in neither the treated nor the control group), we cannot rule out that the observed attenuation of LV dilation may be induced by the beta-blockade effect of amiodarone. Importantly, from a safety standpoint, no increase in myocardial fibrosis was observed after long-term amiodarone administration.

## Conclusions

Long-term experimental oral administration of amiodarone in doses similar to the ones used in the clinical setting was not associated with adverse effects on myocardial fibrosis and other features of adverse cardiac remodeling. Given the promising results of amiodarone effectiveness in suppressing cardiac arrhythmias, these new safety findings suggest that anti-arrhythmic therapy with amiodarone warrants further clinical investigation in the subpopulation of heart failure patients with increased burden of supraventricular or life-threatening ventricular arrhythmias.
